# Association of *Gremlin-2* gene polymorphisms with osteoporosis risk in Chinese postmenopausal women

**DOI:** 10.1042/BSR20200554

**Published:** 2020-04-24

**Authors:** Yu Feng, Lei Zhu, Yong Gu, Ling-Jun Wang, Bing-Jie Niu, Feng Cai, Liang Chen

**Affiliations:** 1 Department of Orthopaedics, The First Affiliated Hospital of Soochow University, Suzhou, China; 2 Department of Spine Surgery, Zhongda Hospital, School of Medicine, Southeast University, Nanjing, China

**Keywords:** Case–control study, GREM2, osteoporosis, postmenopausal women

## Abstract

The Gremlin-2 (GREM2) plays crucial roles in modulating bone homeostasis through the bone morphogenetic protein-2 pathway. However, *GREM2* gene variants in osteoporosis were less frequent in a Chinese population. Therefore, the present study recruited 310 patients with osteoporosis and 339 healthy postmenopausal women to assess the correlation of *GREM2* gene polymorphisms with the risk of osteoporosis. Polymerase chain reaction (PCR) and Sanger sequencing were utilized to genotype samples. The results showed that *GREM2* gene rs4454537, not rs11588607, polymorphism was significantly associated with an increased risk of osteoporosis in postmenopausal women. Moreover, stratified analyses indicated a significant association between rs4454537 polymorphisms and body mass index of <25 kg/m^2^. Additionally, the association between *GREM2* rs4454537 polymorphism and clinical characteristics was assessed, which showed that this locus decreased the bone mineral density (BMD) in postmenopausal osteoporotic individuals. Furthermore, individuals with CC genotype appeared to have a higher GREM2 expression compared with those bearing the TT genotype of rs4454537 polymorphism. However, the genotype distribution of rs4454537 polymorphism showed no statistical difference between osteoporotic patients as a function of fracture status. In summary, *GREM2* rs4454537 polymorphism decreases BMD and increases osteoporotic risk in postmenopausal women.

## Introduction

Osteoporosis is a systemic condition wherein patients exhibit low bone mass and microstructural degeneration of osseous tissue, resulting in increased bone fragility and fracture susceptibility [1]. The mean prevalence of osteoporosis in older adults was about 15.7% and it is reported to be 202.43 million people in China [2]. This condition is most common among women, who account for >80% of cases, with incidence being particularly high in women after menopause [3,4]. Many factors, including loss of bone mineral density (BMD), vitamin D deficiencies, estrogen deficiency, environmental factors, and several genetic factors can drive postmenopausal osteoporosis [5]. Cheung et al. found that Gremlin-2 (GREM2) regulated osteoblasts differentiation and osteogenesis [6,7] and its polymorphism was related to low BMD [8].

GREM2 is a key bone morphogenetic protein (BMP) antagonist [9]. GREM2, as a BMP antagonist, competes with the BMP receptor to regulate the activity of BMP ligands [10,11]. BMP activity changes within fully developed bone are linked to osteoporosis, osteoarthritis, and decreased bone fracture healing capacity [12]. Additionally, previous studies have shown that GREM2 is regulated by the Wnt/β-catenin signaling pathway; it participates in osteoblast differentiation and is involved in osteogenesis [6,7]. Thus, it is believed that GREM2 might be a prominent candidate gene for susceptibility to osteoporosis.

*GREM2* gene variants have been studied in various diseases [13,14], but rarely in osteoporosis. Kaminski et al. reported no significant association between *GREM2* rs4454537 polymorphism and osteoporosis risk in Polish postmenopausal women [15]. This finding has not been confirmed in a Chinese population. Furthermore, *GREM2* rs11588607 and rs4454537 polymorphisms were related to a low femoral neck BMD and whole hip BMD, respectively, and rs4454537 was successfully replicated in an independent cohort [8]. The accelerated loss of hip BMD could contribute to osteoporotic fracture [16]. Therefore, this case–control study was designed as a means of exploring how GREM2 gene polymorphisms impact osteoporosis susceptibility, BMD levels, and fracture incidence.

## Materials and methods

### Subjects

Totally, we recruited 649 unrelated Chinese postmenopausal women from the First Affiliated Hospital of Soochow University, including 310 osteoporosis patients and 339 healthy postmenopausal women. Osteoporosis was diagnosed based upon World Health Organization criteria. The inclusion criteria consisted of an age range of 40–90 years, reaching the menopause at least one year ago, no hormone replacement therapy, or drugs affecting bone mass. Patients with an unbalanced thyroidal condition, kidney failure, malabsorption syndrome, and active neoplastic disease were not included in the present study. During a year of follow-up, 181 of 310 osteoporosis patients were found to have fractured. Among the 181 fracture cases, 54 (29.8%) were lumbar spine compression fractures, 36 (19.9%) were intertrochanteric fractures, 36 (19.9%) were distal radius fractures, 22 (12.2%) were thoracic compression fractures and 33 (18.2%) were ankle fractures. The control group consisted of 339 postmenopausal women with the same age range in the same region. The subjects were interviewed using a standard questionnaire to collect data on demographics, BMI, age at menopause, and medical history. All the subjects submitted informed consent forms before enrollment. The study protocol was confirmed by the Ethics Committee of the First Affiliated Hospital of Soochow University and performed according to the guidelines of the Helsinki Declaration.

### BMD measurement

Two radiologists assessed total hip BMD and BMD at the lumbar spine (L_2_–L_4_), with these radiologists being blinded to the other medical data. A dual-energy X-ray absorptiometer (Lunar Co., WI, U.S.A.) was used for these measurements. The coefficients of variation of the BMD values were 0.2%. BMDs were recorded as g/cm^2^ and a percentage of peak bone mass in the controls (*T*-score). Based on these measurements, the subjects were classified into the following categories: osteopenic (*Y* score between -2.5 and -1), osteoporotic (*T*-score under -2.5), and healthy groups (*T*-score over -1).

### Blood sampling and genotyping

A 2-ml peripheral blood sample was collected from all the participants, and genomic DNA was extracted from the whole blood with a TIANamp blood DNA kit (Tiangen Biotech, Beijing, China) based on provided directions, followed by storage at −20°C for further tests. Genotyping of GREM2 rs11588607 and rs4454537 polymorphisms was conducted via polymerase chain reaction (PCR) and Sanger sequencing techniques. The specific primers were rs4454537: 5′-TCTGTATTGGGCTGTTGT-3′ (F) and 5′-CTGCTTAATTTGGTGGGT-3′ (R); rs11588607: 5′-CTTTAGGTTTGGGTTGAT-3′ (F) and 5′-TAGCCTTGCTTTACTTCT-3′ (R). The PCR products were sent to Genscript. Inc (Nanjing, Jiangsu, China) for Sanger sequencing procedure. Approximately 10% of the samples of all the subjects underwent repeated genotyping, and the genotypes were 100% concordant.

### Real-time PCR analysis

Total RNA was extracted from whole blood using the Trizol reagent (Invitrogen). Real-time PCR was performed after reverse transcription with a Roche LC 480 system with a QuantiTect SYBR Green RT-PCR kit (Qiagen, Hilden, Germany). Glyceraldehyde 3-phosphate dehydrogenase (GAPDH) was included as the internal control. Data were analyzed by Ct (2^−ΔΔCt)^ method and expressed as the fold change compared with GAPDH. Primers were used for RT-PCR were as follows: GREM2, 5′-AGAGTGACTGGTGCAAGACG-3′ (forward) and 5′-TGATTCGGAAAGGTGGGTCG-3′ (reverse); and GAPDH, 5′-GTTCCAATATGATTCCACCC-3′ (forward) and 5′- AGGGATGATGTTCTGGAGAG-3′(reverse).

### Statistical analyses

All the data were tested on Statistical Product and Service Solutions (SPSS) 22.0 (IBM, Chicago, U.S.A.). The Student’s *t*-test or chi-squared (χ2) test were used for comparing demographic and clinical differences between the cases and control groups. A χ2 analysis was used to evaluate whether the genotype distribution of healthy controls conformed to the Hardy–Weinberg equilibrium (HWE). Stratified analyses were conducted in terms of drinking, smoking, BMI, and age. The correlation between GREM2 gene polymorphisms and the risk of osteoporosis was assessed via logistic regression by calculating the odds ratios (ORs) and 95% confidence intervals (CI). *P* < 0.05 indicated a significant difference.

## Results

### Characteristics of the study population

The details of all the subjects are summarized in Table 1. The patients and controls were 62.77 and 62.79 years of age on average, respectively. No significant inter-group differences were identified in age, BMI, and serum phosphorus levels. In addition, the distributions of smokers and drinkers in the case group were comparable to those in the control group, whereas serum calcium, L_2_-L_4_ BMD, and *T*-scores were significantly lower in the osteoporotic patients versus controls (*P*<0.05).

**Table 1:** The general characteristics of study participants

Variable	Cases (*n*=310)	Controls (*n*=339)	*P*
Age (years)	62.77 ± 8.42	62.79 ± 8.30	0.980
BMI (kg/m^2^)	24.40 ± 1.44	24.26 ± 1.52	0.230
Serum calcium (mg/dl)	9.31 ± 0.65	9.55 ± 0.57	<0.001
Serum phosphorus (mg/dl)	4.55 ± 2.76	4.32 ± 3.01	0.327
Smoking (%)	25 (8.1%)	23 (6.8%)	0.702
Drinking (%)	61 (19.7%)	58 (17.1%)	0.406
Fracture (%)	181 (58.4%)		
BMD L2-L4 (g/cm^3^)	0.73 ± 0.06	1.01 ± 0.06	<0.001
*T*-score	−3.19 ± 0.39	0.12 ± 0.10	<0.001

Abbreviations: BMD, bone mineral density; BMI, body mass index.

### The analysis of GREM2 gene variants

Table 2 presents the genotypes and allele distributions of *GREM2* rs11588607 and rs4454537 polymorphisms. The HWE testing revealed no obvious deviation in genotypic frequency in the controls, indicating that these subjects were representatives of the local population. *GREM2* rs11588607 polymorphism exhibited negative correlation with the risk of postmenopausal osteoporosis under five models. Logistic regression analyses showed that CC genotype of rs4454537 polymorphism markedly elevated the risk of osteoporosis (CC vs. TT: OR = 1.85, 95% CI: 1.01–3.38, *P*=0.047) (Table 2). Furthermore, the C allele of rs4454537 polymorphism was correlated with a higher risk of osteoporosis (C vs. T: OR = 1.29, 95% CI: 1.01–1.65, *P*=0.041) (Table 2). Subgroup analyses were conducted in terms of age, BMI, smoking, and drinking status. No significant association was observed with the risk of osteoporosis in any subgroup for rs11588607. However, there was a significantly higher risk of osteoporosis in BMI < 25 kg/m^2^ (Table 3).

**Table 2:** The association of genotype and allele of GREM2 rs11588607/rs4454537 polymorphism with osteoporosis risk

Genotype	Genotypes and alleles	Frequencies, *N*(%)		OR (95% CI)	*P*
		Cases (*n*=310)	Controls (*n*=339)		
Rs11588607					
	CC	145 (46.8%)	169 (49.9%)	1.0	
	CT	133 (42.9%)	143 (42.2%)	1.08 (0.78,1.50)	0.626
	TT	32 (10.3%)	27 (8.0%)	1.38 (0.79,2.41)	0.257
	TT+CT	165 (53.2%)	170 (50.1%)	1.13 (0.83,1.54)	0.433
	CC+TT	278 (89.7%)	312 (92.0%)	1.0	
	TT	32 (10.3%)	27 (8.0%)	1.33 (0.78,2.28)	0.298
	C allele	423 (68.2%)	481 (70.9%)	1.0	
	T allele	197 (31.8%)	197 (29.1%)	1.14 (0.90,1.44)	0.288
Rs4454537					
	TT	156 (50.3%)	192 (56.6%)	1.0	
	TC	124 (40.0%)	127 (37.5%)	1.20 (0.87,1.66)	0.268
	CC	30 (9.7%)	20 (5.9%)	**1.85 (1.01,3.38)**	**0.047**
	CC+TC	154 (49.7%)	147 (43.4%)	1.29 (0.95,1.76)	0.107
	TC+TT	280 (90.3%)	319 (94.1%)	1.0	
	CC	30 (9.7%)	20 (5.9%)	1.71 (0.95,3.08)	0.074
	T allele	436 (70.3%)	511 (75.4%)	1.0	
	C allele	184 (29.7%)	167 (24.6%)	**1.29 (1.01,1.65)**	**0.041**

Bold values are statistically significant (*P*<0.05).

**Table 3:** Stratified analyses between rs11588607/rs4454537 polymorphisms and the risk of osteoporosis

Variable	Case/Control			Heterozygous	Homozygous	Recessive	Dominant
	CC	CT	TT	CT vs. CC	TT vs. CC	TT vs. CT+CC	TT+CT vs. CC
rs11588607							
Age							
<60 years	55/65	49/60	13/10	0.94 (0.55,1.60); 0.815	1.40 (0.57,3.44); 0.470	1.44 (0.61,3.42); 0.412	1.01 (0.61,1.67); 0.976
≥60 years	90/104	84/83	19/17	1.18 (0.78,1.78); 0.428	1.37 (0.67,2.78); 0.390	1.27 (0.64,2.51); 0.500	1.21 (0.82,1.79); 0.337
BMI							
<25 kg/m^2^	96/117	87/98	24/20	1.08 (0.73,1.61); 0.696	1.46 (0.76,2.81); 0.253	1.41 (0.75,2.64); 0.282	1.15 (0.79,1.67); 0.474
≥25 kg/m^2^	49/52	46/45	8/7	1.09 (0.62,1.91); 0.778	1.21 (0.41,3.60); 0.728	1.17 (0.41,3.34); 0.774	1.10 (0.64,1.90); 0.727
Smoking							
No	131/155	124/137	30/24	1.07 (0.77,1.50); 0.690	1.48 (0.82,2.65); 0.190	1.43 (0.82,2.51); 0.212	1.13 (0.82,1.56); 0.450
Yes	14/14	9/6	2/3	1.50 (0.42,5.35); 0.532	0.67 (0.10,4.62); 0.682	0.58 (0.09,3.83); 0.571	1.22 (0.39,3.86); 0.733
Drinking							
No	117/143	105/115	27/23	1.12 (0.78,1.60); 0.551	1.44 (0.78,2.63); 0.244	1.36 (0.76,2.45); 0.298	1.17 (0.83,1.65); 0.370
Yes	28/26	28/28	5/4	0.93 (0.44,1.96); 0.846	1.16 (0.28,4.80); 0.837	1.21 (0.31,4.73); 0.789	0.96 (0.47,1.97); 0.906

Variable	Case/Control			Heterozygous	Homozygous	Recessive	Dominant
	TT	TC	CC	TC vs. TT	CC vs. TT	CC vs. TC+TT	CC+TC vs. TT
rs4454537							
Age							
<60 years	55/77	50/51	10/7	1.39 (0.82,2.36); 0.221	2.13 (0.73,6.22); 0.165	1.85 (0.65,5.27); 0.247	1.48 (0.89,2.46); 0.132
≥60 years	99/115	74/76	20/13	1.10 (0.73,1.66); 0.657	1.72 (0.83,3.58); 0.148	1.65 (0.81,3.37); 0.168	1.19 (0.81,1.76); 0.384
BMI							
<25 kg/m^2^	99/135	89/86	19/14	1.41 (0.95,2.09); 0.087	1.85 (0.89,3.87); 0.102	1.60 (0.78,3.27); 0.202	**1.47 (1.01,2.15); 0.044**
≥25 kg/m^2^	57/57	35/41	11/6	0.85 (0.48,1.53); 0.594	1.83 (0.64,5.29); 0.263	1.95 (0.69,5.50); 0.205	0.98 (0.57,1.69); 0.939
Smoking							
No	145/182	113/115	27/19	1.23 (0.88,1.73); 0.226	1.78 (0.95,3.34); 0.070	1.64 (0.89,3.01); 0.114	1.31 (0.95,1.81); 0.099
Yes	11/10	11/12	3/1	0.83 (0.26,2.72); 0.763	2.73 (0.24,30.66); 0.416	3.00 (0.29,31.11); 0.357	0.98 (0.31,3.07); 0.971
Drinking							
No	129/160	100/108	20/13	1.15 (0.80,1.64); 0.448	1.91 (0.91,3.98); 0.085	1.80 (0.88,3.70); 0.110	1.23 (0.87,1.73); 0.237
Yes	27/32	24/19	10/7	1.50 (0.68,3.30); 0.317	1.69 (0.57,5.05); 0.345	1.43 (0.50,4.05); 0.502	1.55 (0.75,3.19); 0.235

BMI, body mass index

Bold values are statistically significant (*P*<0.05).

Next, the clinical and biochemical characteristics of *GREM2* rs4454537 polymorphism were compared between the osteoporotic patients and the healthy controls (Table 4). There were no significant differences between the three genotypes of rs4454537 polymorphism in terms of age, BMI, serum calcium and phosphorus levels, and *T*-scores. However, for osteoporosis, the L_2_-L_4_ BMD of the CC genotype was significantly lower than the TT genotype, indicating that *GREM2* rs4454537 polymorphism showed a significant correlation with L_2_-L_4_ BMD.

**Table 4:** The clinical and biochemical characteristics of GREM2 rs4454537 polymorphism among two groups

Variables	TT	CT	CC	*P*
**Osteoporosis**				
Age (years)	63.09 ± 8.55	62.18 ± 8.07	63.57 ± 9.30	0.576
BMI (kg/m^2^)	24.54 ± 1.44	24.20 ± 1.35	24.50 ± 1.78	0.127
Serum calcium (mg/dL)	9.32 ± 0.66	9.30 ± 0.65	9.29 ± 0.56	0.954
Serum phosphorus (mg/dL)	4.73 ± 3.42	4.44 ± 1.96	4.06 ± 1.51	0.407
BMD L2-L4 (g/cm^2^)	0.74 ± 0.067	0.72 ± 0.063	0.72 ± 0.055	**0.012**
*T*-score	-3.14 ± 0.37	-3.25 ± 0.39	-3.17 ± 0.43	0.051
**Control**				
Age (years)	62.81 ± 8.58	62.42 ± 7.86	64.90 ± 8.33	0.462
BMI (kg/m^2^)	24.22 ± 1.51	24.29 ± 1.54	24.42 ± 1.57	0.806
Serum calcium (mg/dL)	9.58 ± 0.56	9.53 ± 0.59	9.41 ± 0.44	0.383
Serum phosphorus (mg/dL)	4.56 ± 3.29	3.99 ± 1.98	4.18 ± 5.05	0.254
BMD L2-L4 (g/cm^2^)	1.01 ± 0.055	1.01 ± 0.055	1.00 ± 0.069	0.566
*T*-score	0.10 ± 0.01	0.10 ± 0.10	0.09 ± 0.01	0.688

Bold values are statistically significant (*P*<0.05).

### *GREM2* mRNA expression in different genotype

**Figure 1:**
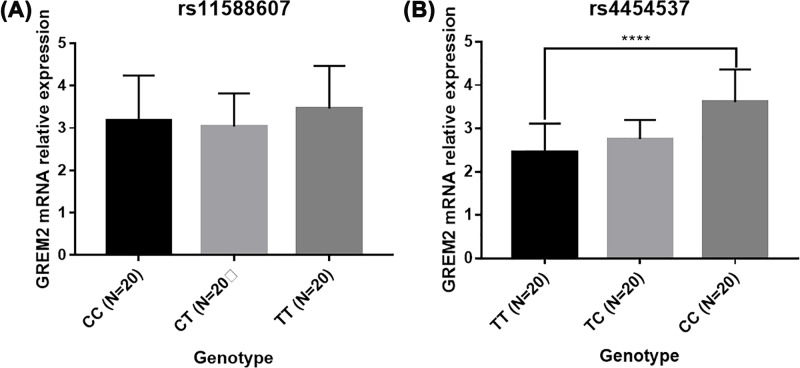
GREM2 mRNA expression in different genotype (**A**) rs11588607; (**B**) rs4454537. ****P<0.05.

Furthermore, we evaluated the effect of *GREM2* gene polymorphisms on the GREM2 expression. For rs11588607, there is no significant difference among three genotypes with regard to the GREM2 levels (Figure 1A). However, our results indicated that the up-regulation of *GREM2* were observed in CC genotype than those in TT genotype of rs4454537 polymorphism (Figure 1B).

### Association of GREM2 rs4454537 polymorphism with fracture risk

The present study also investigated whether *GREM2* rs4454537 polymorphism was linked to the risk of osteoporotic fracture since this polymorphism conferred susceptibility to osteoporosis (Table 5). The genotype distribution of rs4454537 polymorphism was statistically comparable between osteoporotic patients with and without fracture, indicating no statistical correlation with fracture.

**Table 5:** The genotype and allele frequency distribution of rs4454537 polymorphism in postmenopausal osteoporosis patients with and without fracture

Genotypes and alleles	Frequencies, *N* (%)		OR (95% CI)	*P*
	Without fracture (*n*=129)	With fracture (*n*=181)		
TT	68 (52.7%)	88 (48.6%)	1.0	
TC	45 (34.9%)	79 (43.6%)	1.36 (0.84,2.20)	0.216
CC	16 (12.4%)	14 (7.7%)	0.68 (0.31,1.48)	0.326
CC+TC	61 (47.3%)	93 (51.4%)	1.18 (0.75,1.85)	0.477
TC+TT	113 (87.6%)	167 (92.3%)	1.0	
CC	16 (12.4%)	14 (7.7%)	0.59 (0.28,1.26)	0.171
T allele	181 (70.2%)	255 (70.4%)	1.0	
C allele	77 (29.8%)	107 (29.6%)	0.99 (0.70,1.40)	0.939

## Discussion

Our findings revealed that *GREM2* rs4454537, not rs11588607, polymorphism increased osteoporosis risk in Chinese postmenopausal women, especially in subjects with BMI < 25 kg/m^2^. In addition, this polymorphism was linked to reduced BMD in those with osteoporosis. In contrast, the rs4454537 polymorphism allele and genotype distributions were comparable between cases and controls.

The BMP-2 pathway plays a vital role in positively modulating bone homeostasis. The suppression of BMP antagonist GREM2 increased the BMP-2-induced osteogenesis of human bone marrow-derived mesenchymal stem cells (BMSCs) [17]. *GREM2* gene variants might influence the expression and function of GREM2. Several recent studies have addressed the relationship between *GREM2* gene polymorphism and the risk of osteoporosis [8,15]. Cheung et al. were the first researchers to investigate the relationship between *GREM2* rs11588607 and rs4454537 polymorphisms and areal BMD in a southern Chinese population with 417 cases and 359 controls [8]. They observed that *GREM2* rs11588607 and rs4454537 were linked to a low BMD at the femoral neck, spine, and total hip [8].

Furthermore, the positive result of rs4454537 was repeated in an additional 454 cases and 401 controls [8]. Later, a Polish cohort with 333 osteoporosis patients and 233 healthy women exhibiting the genotype distribution of the *GREM2* gene showed no significant difference between groups [15]. Herein, *GREM2* rs4454537 polymorphism increased osteoporotic risk in the Chinese postmenopausal women evaluated, with 310 cases and 339 controls; however, this strong correlation did not apply to rs11588607 polymorphism. This significant association also appeared to be strong in people with BMI < 25 kg/m^2^. Furthermore, the mutant genotype of rs4454537 polymorphism showed a significant correlation with a decreased BMD, while it did not confer susceptibility to fracture.

To sum up, a significant association was found between *GREM2* rs45454537 polymorphism and the risk of osteoporosis, which is different from the results of a study by Kaminski et al. Many factors might account for the contradictory results. First, different races and clinical heterogeneity might cause different genotype frequencies of rs4454537 polymorphism in the Polish [15] and Chinese populations. Second, different sample sizes should also be taken into consideration. Additionally, *GREM2* rs4454537 polymorphism was found to be associated with a decreased BMD in Hong Kong [8] and mainland China (the present study) populations. In addition, the role of this polymorphism in the fracture risk was investigated, which is different from the results of a study by Cheung [8]. Although *GREM2* rs4454537 polymorphism decreased BMD, it was not associated with the risk of fracture. We could not rule out the possibility of false negative results due to the limited sample size.

The present study has certain limitations. First, the study was hospital-based; therefore, selection bias was inevitable. Second, the gene–environment interactions were not investigated in the present study, including gene–diet and gene–physical activity variables. Three, the medium sample size might make the study underpowered. Fourth, the GREM2 expression levels in the three genotypes of rs4454537 polymorphism were not measured, which was a serious limitation.

It was concluded that *GREM2* rs4454537 polymorphism decreases the BMD and increases osteoporotic risk among postmenopausal women. Studies of other Chinese populations are urgently needed to explore the relationship between this polymorphism and postmenopausal susceptibility to osteoporosis.

## Abbreviations

BMD: bone mineral density
CI: confidence interval
GREM2: Gremlin-2
OR: odds ratio
PCR: polymerase chain reaction
